# The Anisotropic Osteoinductive Capacity of a Nickel–Titanium Alloy Fabricated Through Laser Powder Bed Fusion

**DOI:** 10.3390/ijms26104640

**Published:** 2025-05-13

**Authors:** Yu Sun, Zhenglei Yu, Qingping Liu, Luquan Ren, Xin Zhao, Jincheng Wang

**Affiliations:** 1 Orthopaedic Medical Center, The 2nd Hospital of Jilin University, Changchun 130041, China; yusun@jlu.edu.cn; 2 Key of Bionic Engineering, Ministry of Education, Jilin University, Changchun 130022, China; zlyu@jlu.edu.cn (Z.Y.); liuqp@jlu.edu.cn (Q.L.); lqren@jlu.edu.cn (L.R.)

**Keywords:** LPBF-NiTi, osteoinductive capacity, anisotropy, biofixation, orthopedic implants

## Abstract

A novel parameter optimization method for additively manufacturing nickel–titanium (NiTi) alloys using laser powder bed fusion (LBPF) was developed. Compared with the conventional NiTi alloy and the previously reported LPBF-NiTi alloy, the LBPF-NiTi alloy prepared with these parameters exhibits excellent tensile properties and an anisotropic microstructure. Since distinct regions of orthopedic implants have specific functional requirements, we investigated the anisotropy of this LPBF-NiTi in terms of its osteoinductive capacity to determine the appropriate building direction for prosthesis fabrication. The biosafety of the transverse (XY-NiTi) and longitudinal (XZ-NiTi) planes was assessed through cytotoxicity assays. Comparative analyses of the biological activities of these planes were conducted by evaluating the adherent cell counts, the adhesion morphology, and the expression of osteogenic-related genes and factors in adherent cells. Compared with XZ-NiTi, XY-NiTi exhibited superior cell adhesion properties. Additionally, the expression levels of osteogenic markers (RUNX2, ALP, OPG, and OCN) were significantly greater in bone marrow mesenchymal cells (BMMCs) adhered to XY-NiTi than in those adhered to XZ-NiTi. These results indicate a greater osteogenic potential in the XY-NiTi group. XY-NiTi was more advantageous as an implant–bone contact surface. Building implant products in the direction perpendicular to the load-bearing axis enhances biofixation; thus, this is the preferred orientation for manufacturing orthopedic implants.

## 1. Introduction

The demand for orthopedic implants has increased dramatically, and predictions indicate that by 2030, the annual number of total hip joint surgeries in the United States alone will reach 4 million [1]. Despite various efforts to enhance the biological fixation of biocompatible orthopedic implants, the failure rate of hip implants remains high, necessitating revision surgeries that cause additional pain and financial burdens for patients [2,3]. The primary cause of these failures is bone resorption due to stress shielding [3,4,5,6,7], which arises from the mismatch in the elastic modulus between the prosthesis and the surrounding bone.

Nitinol shape memory alloy (NiTi-SMA) is the most widely used SMA and is renowned for its excellent functional properties. NiTi is a promising material for a range of medical devices, including scaffolds, compression screws, femoral stems, and self-expanding acetabular prostheses [8]. NiTi alloys are particularly suitable for orthopedic implants based on the following two key properties: (1) a lower modulus of elasticity (approximately 95 GPa for conventional NiTi) [9,10,11,12] than Ti-6Al-4V (~110 GPa) and (2) unique superelasticity and shape memory capabilities, which theoretically enable a better press fit.

However, the high reactivity and low thermal conductivity of NiTi SMAs cause significant challenges in the subsequent processing of their preliminarily formed parts [13,14]. Traditional manufacturing processes are restricted to molding and subtractive techniques, limiting the ability to create NiTi components with complex geometries, such as porous structures, negative-Poisson-ratio designs, and layered configurations.

In the past decade, laser powder bed fusion (LPBF) has garnered significant attention for processing NiTi alloys, primarily because of its ability to overcome many challenges associated with traditional manufacturing methods. LPBF-NiTi alloys are known for their excellent compressive properties, since inner defects such as pores and cracks can be effectively closed under compression [13,15,16]. However, the presence of columnar grains in these alloys leads to poor tensile properties [17] because cracks tend to propagate more easily under tensile deformation [18,19,20].

Recent studies by Xiong et al. [17] and Zhang et al. [21] reported significant improvements in the tensile properties of LPBF-NiTi alloys. By modifying the laser scanning length and direction, these researchers were able to eliminate the formation of unidirectional columnar crystals in the material samples, thereby significantly increasing the tensile strain to 15.6%. This value is more than double the previously reported maximum tensile strain of approximately 7% [22,23,24,25,26].

Despite these advancements, the fundamental nature of LPBF involves layer-by-layer laser sintering, which induces localized continuous rapid melting and nonequilibrium solidification. As a result, the microstructure of NiTi fabricated via LPBF differs significantly from that produced through conventional methods. Qiu et al. demonstrated that notable differences were observed in the microstructure and corrosion resistance of LPBF-NiTi depending on the orientation [27]. Specifically, the transverse (XZ) direction and the longitudinal (XY) direction had distinct characteristics. The grain size in the XY direction was smaller than that in the XZ direction; this contributed to a more uniform and dense passivation film on the XY-NiTi.

**Figure 1:**
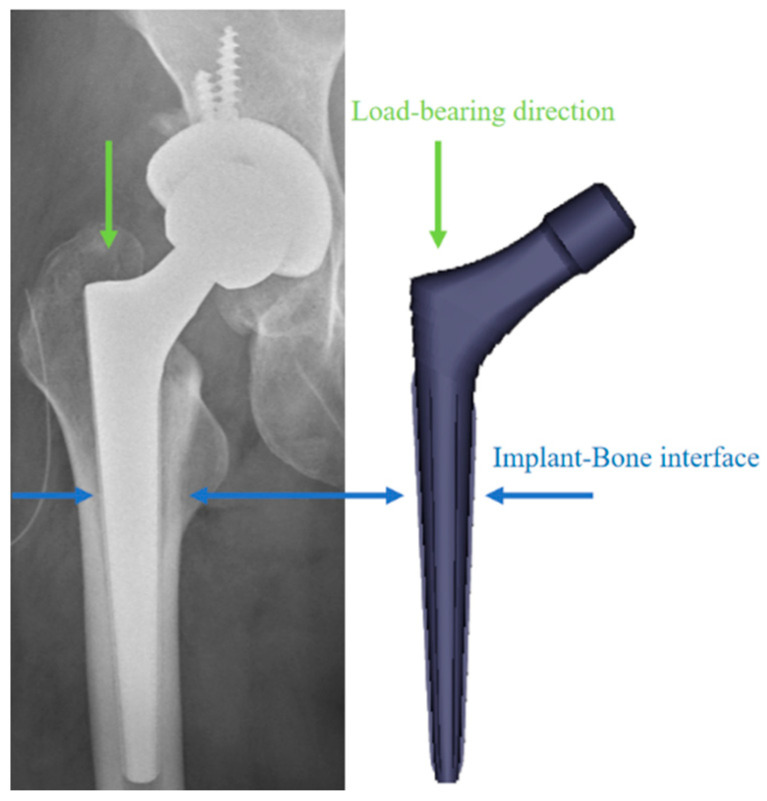
Morphology of the hip prosthesis (SL cone, AK MEDICAL, Beijing, China). The blue arrows represent the implant–bone interface, and the green arrows represent the load-bearing direction.

Grain size is known to significantly impact cell biocompatibility and adhesion behavior when the material composition is consistent. Alloys with finer grains exhibit a greater surface energy, leading to stronger osteoblast adhesion than those with coarser grains [28,29]. As depicted in Figure 1, the prosthesis–bone interface requires an enhanced osteoinductive capacity to improve osseointegration and ensure long-term stability [30,31]. When employing LPBF-NiTi for joint prosthesis fabrication, the biocompatibility and biological activity variations due to the microstructural heterogeneity need to be considered to determine the optimal building direction for the products.

Previous studies predominantly focused on comparing the biocompatibility of LPBF-NiTi with conventional Ti-6Al-4V [32], neglecting the anisotropic interplay between structural, mechanical, and biological properties. In this study, we evaluated the biocompatibility and osteoinductive capacity of XY-NiTi and XZ-NiTi through cytotoxicity assays, adherent cell counting, morphological analyses, and the evaluation of the expression of osteogenic-related genes. The aim was to identify the optimal building direction for implants, with the goal of achieving superior biofixation. However, we acknowledged the limitations of this study, particularly the lack of in vivo studies, and hoped to address this aspect in future research.

## 2. Results

### 2.1. Cytotoxicity

**Figure 2:**
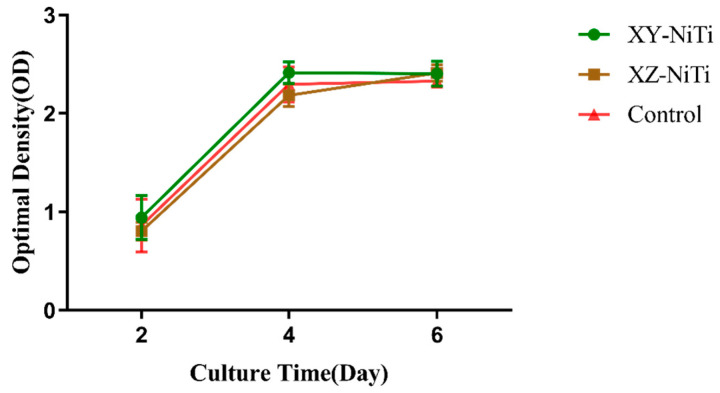
Proliferation of BMMCs cultured in XZ-NiTi and XY-NiTi leaching liquors for two, four, or six days. *n* = 5.

The cytotoxicity assessment of the alloy samples and the viability of the cells surrounding the alloy were assessed via the CCK-8 method. The growth curves of the bone marrow mesenchymal cells (BMMCs) cultured with the two alloy extracts closely resembled those of the cells cultured in a standard medium, as illustrated in Figure 2. Notably, at each time point during the culture period, no significant differences were observed in the number of viable cells among the three groups. Furthermore, the cell viability of both groups of cells treated with the alloys exceeded 90%, as indicated in Table 1.

Remarkably, the cytotoxicity assessment of LPBF-NiTi yielded a rating of level 0–1 according to the ISO 10993-5 standard [33]; this level is commonly interpreted to indicate a safe level of cytotoxicity.

### 2.2. Cell Adhesion Morphology

**Figure 3:**
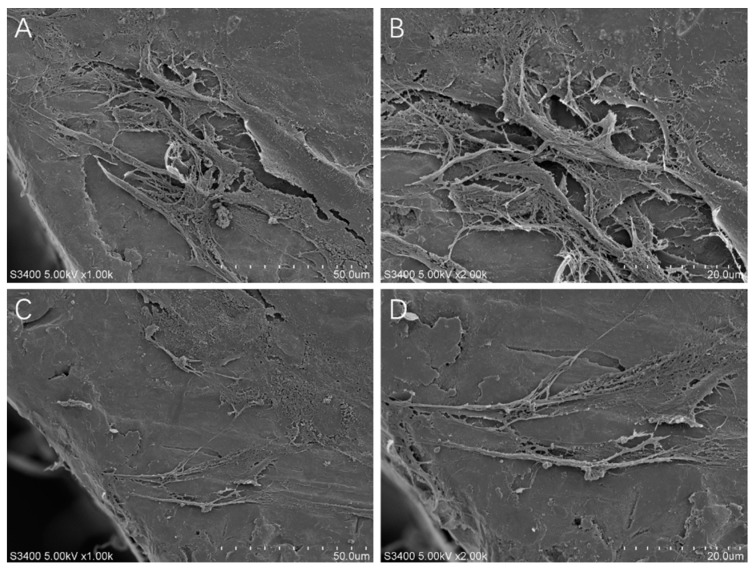
Electron microscopy images of the bone BMMCs cultured on the alloy for 3 days. (**A**,**B**): cells adhered to the XY-NiTi. (**C**,**D**): cells adhered to XZ-NiTi.

After a 3-day culture period on the alloy samples, the adhesion morphology of the BMMCs was observed using scanning electron microscopy. Remarkably, a substantial number of BMMCs adhered to the surface of both groups of alloys. Notably, on XY-NiTi, the cells exhibited a more elongated shape with increased pseudopodia formation, as shown in Figure 3A–D.

### 2.3. Adherent Cell Counting

**Figure 4:**
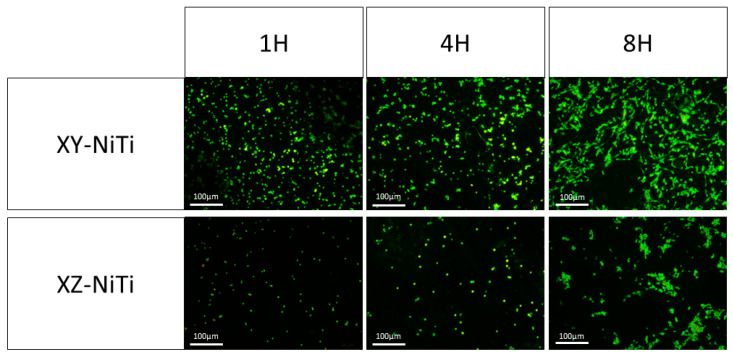
Fluorescence microscope images of acridine orange (green fluorescence = living cells).

**Figure 5:**
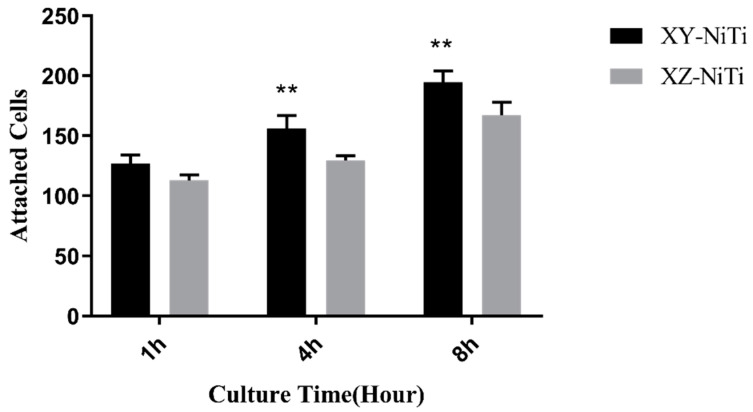
Acridine orange (OA) staining and counting results after BMMC/alloy coculture for one, four, and eight hours. *n* = 3, ** *p* < 0.01.

The ability of the alloys to adhere to cells was assessed via OA staining. Under blue-light excitation, OA-stained living cells emitted green fluorescence, whereas apoptotic cells emitted yellow–green fluorescence. The fluorescence microscope image of the OA-stained BMMCs is depicted in Figure 4; here, all cells exhibited green fluorescence, confirming the viability of the adherent cells. A comparison of the viable cell counts revealed that the number of adherent cells on the surface of XY-NiTi was significantly greater than that on XZ-NiTi (Figure 5). This finding indicated that XY-NiTi had a stronger cell adhesion ability than XZ-NiTi.

### 2.4. Nuclei–Actin–FAK Staining

**Figure 6:**
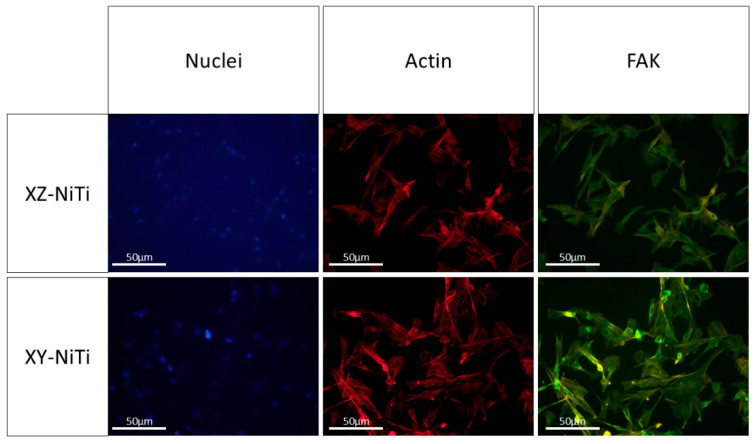
Nuclei, actin, focal adhesion kinase (FAK), and merged images of adherent cells under a fluorescence microscope (blue: nuclei; red: actin; green: FAK).

Nuclei–actin–FAK staining was employed to assess the adhesion state of the cells on the alloy surface. In fluorescence microscopy, nuclei emit blue fluorescence, actin emits red fluorescence, and FAK emits bright green fluorescence. An image of the stained BMMCs is shown in Figure 6. The actin filaments of the BMMCs adhered to XY-NiTi extended radially and traversed the entire cytoplasm; this resulted in a more uniform thickness and distribution than those of the cells adhered to XZ-NiTi. Additionally, the FAK particles appeared uniform in size and were evenly distributed across the surface. The ternary staining results revealed that XY-NiTi possessed superior cell adhesion properties.

### 2.5. Early Osteogenic Differentiation

**Figure 7:**
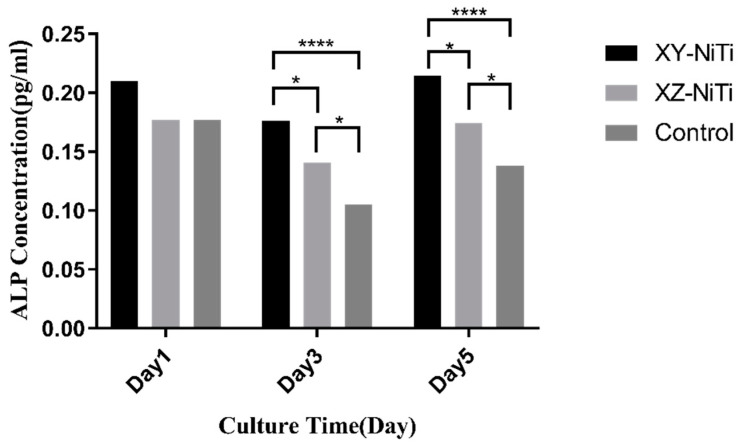
ALP concentrations in the culture medium of the bone marrow mesenchymal cells cultured for one, three, or five days in ordinary medium or medium containing the XZ-NiTi or XY-NiTi alloy. *n* = 6, * *p* < 0.05, **** *p* < 0.0001.

The alkaline phosphatase (ALP) content serves as an early marker of osteoblast differentiation. In this study, the ALP content in the supernatant of the BMMCs cultured with XZ-NiTi, XY-NiTi, or the standard medium was assessed to determine the impact of these materials on the early differentiation of osteoblasts. As depicted in Figure 7, after 1 day of culture, no significant differences were observed among the three groups. However, at 3 and 5 days, ALP secretion from both NiTi alloy groups was notably greater than that from the control group, and the XY-NiTi group exhibited significantly greater ALP secretion than the XZ-NiTi group. These findings indicate that LPBF-NiTi could enhance the osteoblast differentiation, and XY-NiTi showed superior performance between the two alloy groups.

### 2.6. Osteogenic-Related Gene Expression

**Figure 8:**
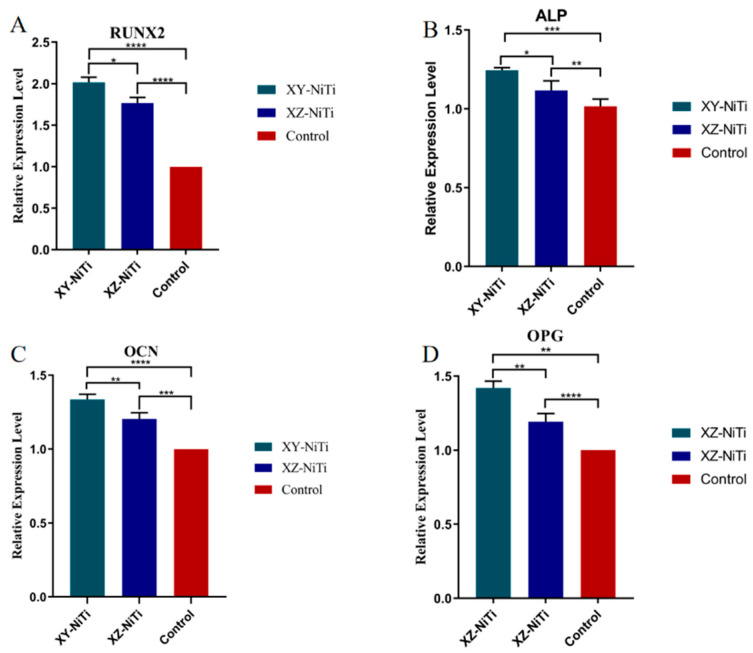
mRNA expression levels of osteogenic genes. (**A**) Runx-2, (**B**) ALP, (**C**) OCN, and (**D**) OPG. *n* = 5, * *p* < 0.05, ** *p* < 0.01, *** *p* < 0.001, and **** *p* < 0.0001.

The expression and secretion of osteogenic proteins by BMMCs play pivotal roles in the integration of implants with bone tissue. To assess the impact of LPBF-NiTi on the expression of osteogenic-related genes, specific genes (ALP, OPG, OCN, and RUNX) were semiquantitatively detected using RT-PCR. Here, XY-NiTi had a significantly greater influence on the expression of ALP (Figure 8); these results were consistent with the observed ALP secretion in the supernatant, as reported in Section 2.5. Furthermore, compared with the control group, both alloy groups demonstrated a significant increase in the expression of OCN, RUNX2, and OPG in BMMCs.

## 3. Discussion

NiTi has good biocompatibility without causing cytotoxic or genotoxic reactions [34]. In this work, we examined the cytotoxicity of two LPBF-NiTi species. As shown in Figure 2, the growth curves of the BMMCs cocultured with the two LPBF-NiTi alloys were similar to those of the control group. Moreover, the relative survival rate of both materials was greater than 90%, demonstrating the absence of cytotoxicity in both materials. These results were consistent with those of previous studies. For example, Habijan et al. [35] studied the response of bone marrow mesenchymal cells (BMMCs) cultured on LPBF-fabricated NiTi. After 8 days of incubation, a substantial number of viable cells were observed on the surface of the implants. Chmielewska et al. [36] studied the biological properties of pre-alloyed and in situ-alloyed LPBF-NiTi with a Ni–Ti ratio of 55.7:44.3 (at. %). The results revealed that the cytotoxicity of both materials was minimal since the cell viability of the L929 cells cultured with the materials was greater than 95%. These studies indicated that the Ni ion release of LPBF-NiTi was drastically below the cytotoxic level. In this work, the nontoxicity of both types of LPBF-NiTi could be attributed to their ability to form protective oxide films [37]; these results were consistent with those from Qiu et al.’s study [27].

Bone marrow cells (BMMCs) play crucial roles in bone tissue repair, and their adhesion to material surfaces is fundamental to their biological function. To evaluate the effect of LPBF-NiTi on osteoblast adhesion, we conducted cell adhesion counts, morphological observations, and cytoskeletal staining of adherent cells. At the same time point, the number of cells adhered to the XY-NiTi surface was significantly greater than that on the XZ-NiTi surface. Furthermore, on the XY-NiTi surface, the BMMCs exhibited a more stretched morphology, more pseudopodia, a more uniform thickness of intracellular actin, and a more uniform distribution of focal adhesion kinase (FAK).

FAK is crucial for the formation and maturation of focal adhesions and promotes their metabolic cycle. Mitra et al. [38] found that the FAK-Src complex regulated the formation and renewal of focal adhesions through the connection of multiple signaling pathways, thereby promoting the migration of normal cells and cancer cells. Ren et al. [39] reported that loss of FAK could activate Rho A, inhibit the renewal of focal adhesions, and inhibit cell migration. Actin filaments play a key role in the early maturation of BMMCs and enhance bone cell functions by promoting cell proliferation and differentiation [40,41]. These findings indicate an enhanced osteogenic differentiation ability and superior cell adhesion on the XY-NiTi surface. Materials with smaller grains are more likely to promote osteoblast adhesion and osteogenic viability in the case of similar compositions [28,29,42]; this potentially explains the superior performance of XY-NiTi in terms of cell adhesion due to its relatively smaller grain size [27].

BMMC adhesion not only anchors cells to the surface of biological materials but also activates cell-signaling pathways, thereby controlling cell differentiation, proliferation, and migration [43]. We analyzed the expression of several proteins and factors related to the differentiation and function of osteoblasts, including ALP, RUNX2, OCN, and OPG. ALP is an early marker of osteoblast differentiation and one of the most important functional genes involved in calcification; it increases inorganic phosphate uptake while decreasing the concentration of extracellular pyrophosphate (which inhibits mineral formation), thereby promoting mineralization [44]. Our results showed that both XY-NiTi and XZ-NiTi promoted the early differentiation of BMMCs into osteoblasts and the mineralization of the osteogenic matrix, and XY-NiTi had a stronger effect. RUNX2 is a major transcription factor for osteoblast differentiation, matrix formation, and mineralization, and can upregulate the expression of ALP and OCN [45]. In our study, RUNX2 expression was significantly greater in the XY-NiTi group than in the XZ-NiTi group; this result explains the greater expression of OCN in the XY-NiTi group. OCN is an osteoblast-secreted protein and a major marker of osteoblast differentiation and maturation [46]. The gene expression results for OCN showed the highest degree of osteogenic differentiation in the XY-NiTi group. OPG competes with RANK to bind RANKL, thereby protecting the bone [47]. Compared with the control group, both the XY-NiTi and XZ-NiTi groups presented significantly greater OPG expression; these results indicated that LPBF-NiTi promoted bone regeneration, and XY-NiTi showed a stronger effect than XZ-NiTi.

Studies have confirmed the anisotropy of the microstructure of Ni-Ti alloys prepared by this method in the XY and XZ planes [27]. The grain size on the XY plane is smaller, and the oxide film is more uniform and denser. Previous studies have confirmed that material surfaces with smaller grain sizes can enhance protein adsorption, which is a key mediator of cell adhesion, function, and tissue growth. The increase in surface area and reactivity is considered the primary reason for this increase [48]. Li et al. [49] compared their developed nanocrystalline Ti_49.2_Ni_50.8_ with commercial microcrystalline Ti_49.2_Ni_50.8_ and found that the nanocrystalline Ti_49.2_Ni_50.8_, with smaller grain sizes, had enhanced cell viability, protein adhesion, proliferation, alkaline phosphatase (ALP) activity, mineralization, and osseointegration. The anisotropic properties of this LPBF-NiTi have significant implications for the design and manufacturing of orthopedic implants. The superior biological activity of XY-NiTi indicates that implant surfaces in contact with bone should be oriented in the XY direction to enhance biofixation. This directional choice can lead to improved long-term stability and functionality of orthopedic implants, reducing the need for revision surgeries and improving patient outcomes.

While this study provides important insights, several limitations should be acknowledged. First, this study revealed that XY-NiTi had better biological activity than XZ-NiTi; however, the underlying mechanisms of this phenomenon have not been investigated. Further research is needed to explore these mechanisms to provide a theoretical basis for further optimizing this alloy. Second, the focus on short-term in vitro assays limits the understanding of the long-term biocompatibility and performance of LPBF-NiTi implants in vivo. Future studies need to include long-term animal studies to evaluate the biocompatibility and mechanical stability of these implants over extended periods. Third, the focus of this study was on the anisotropic biological activity of LPBF-NiTi in the transverse (XZ) and longitudinal (XY) planes; however, the developed LPBF-NiTi alloy was not compared with other alloys. Although the results indicate that XY-NiTi is more suitable as a bone contact surface, the lack of lateral comparison does not demonstrate that this LPBF-NiTi is superior for use as a bone implant. Therefore, future research needs to include comparisons with Ti-6Al-4V and conventionally processed NiTi to further assess the potential of LPBF-NiTi for use in bone implants.

## 4. Materials and Methods

### 4.1. Production of LPBF-NiTi Specimens

The LPBF-NiTi alloy specimens were prepared as previously described [17]. Briefly, gas-atomized NiTi powder (15–53 μm in diameter, provided by Dr. Hao [17,27]) was used to produce LPBF-NiTi (Ti_49.6_Ni_50.4_). LPBF processing was carried out under an argon atmosphere on a NiTi substrate maintained at 180 °C. LPBF-fabricated NiTi (Ti_49.6_Ni_50.4_) was prepared by an LPBF machine (M100-T, Eplus, Beijing, China) equipped with a Yb-fiber laser with a maximum power of 200 W. The parameters used for preparing the samples were as follows: a laser power of 120 W, a laser scanning speed of 500 mm/s, a layer thickness of 30 μm, and a hatch spacing of 80 μm [17,21]. The phase transformation temperature, phase composition, and microstructure were described by Xiong et al. [17]. The LPBF-NiTi exhibited a tensile strain of up to 15.6% and a tensile strength of 700 MPa. XY-NiTi and XZ-NiTi were prepared as disc-shaped samples with a diameter of 20 mm and a thickness of 2 mm. The surfaces and sides of all specimens were mechanically polished to a surface roughness of 0.25 μm, ultrasonically cleaned with distilled water, an acetone solution, and 70% ethanol for 20 min, ultrasonicated with distilled water for 15 min, autoclaved at 121 °C for 40 min, and vacuum-dried.

### 4.2. Isolation of Mouse Bone Marrow Mesenchymal Cells

All experiments were performed in accordance with the relevant guidelines and regulations. Eight-week-old C57BL/6 male mice were sacrificed by an overdose of 3% pentobarbital. The femurs of the sacrificed animals were obtained. The medullary cavity was washed repeatedly with prechilled medium, and the wash solution was collected and centrifuged at 1500 rpm for 5 min. The supernatant was discarded, and the pellet was resuspended in 5 mL of high-glucose Dulbecco’s modified Eagle’s medium (DMEM, Gibco, Waltham, MA, USA) containing 10% fetal bovine serum (FBS, Gibco, Waltham, MA, USA) and then incubated overnight at 5% CO_2_ and 37 °C. The supernatant was subsequently removed, and the bottom was rinsed twice with phosphate-buffered saline (PBS, Coolabar, Beijing, China). The medium was added and changed every 2 days. When 85% confluence was reached, the cells were passaged. The third generation of cells was obtained for study. This study was approved by the Animal Experiment Ethics Committee of Jilin University (approval number: KT202003017), and the authors complied with the ARRIVE guidelines.

### 4.3. Cytotoxicity and Viability

In vitro cytotoxicity and cell viability analyses of the alloy samples were performed using the Cell Counting Kit 8 (CCK-8, Beyotime, Nantong, China) method. The XY-NiTi and XZ-NiTi samples were immersed in acetone and ethanol for 20 min, respectively, and then autoclaved at 0.15 MPa and 121 °C for 40 min. The two alloys were soaked in a culture medium at a ratio of 3 mL/cm^2^ at 37 °C for 24 h to prepare the leaching solution. Bone marrow mesenchymal cells (BMMCs) were suspended in two extracts and then seeded in 96-well plates (200 μL per well) at a concentration of 5 × 10^6^/mL. In addition, BMMCs cultured in a standard medium were used as the control group, and those cultured in a cell-free standard medium were used as the blank group. Five technical replicates were set up per experimental group. On days two, four, and six, 20 μL of the CCK-8 solution was added to each well at 5% CO_2_ and 37 °C, and the mixture was incubated for 4 h. The absorbance value of each well was detected with a spectrophotometer at 450 nm. The cell viability was calculated using the following formula:

$$Cell\;viability=\frac{{OD}_{E}}{{OD}_{C}}$$

where OD_E_ is the optical density of the test group, and OD_C_ is the optical density of the control group. All experiments were performed in five replicates.

### 4.4. Cell Adhesion

Cell adhesion was assessed using acridine orange (OA; Sigma-Aldrich, St. Louis, MO, USA) staining. The mouse BMMCs were inoculated at 1 × 10^6^/mL in six-well plates containing XY-NiTi or XZ-NiTi (3 mL per well). After incubation at 37 °C for 1 h, 4 h, and 8 h, the alloy samples were removed, washed 3 times with PBS, and fixed in 95% ethanol for 15 min. After drying, the samples were soaked in 1% acetic acid for 30 s and stained with 0.01% OA for 1 min. After 3 rinses with PBS, the samples were soaked in 0.1 M CaCl_2_ for 2 min, rinsed again 3 times with PBS, and subjected to fluorescence microscopy (IX51, Olympus, Tokyo, Japan). Five high-magnification fields were subsequently selected to count the number of adherent cells. Each experiment was repeated three times.

### 4.5. Adherent Cell Morphology

BMMCs were seeded at a concentration of 1 × 10^7^/mL in six-well plates containing XY-NiTi or XZ-NiTi (3 mL per well). After 3 days of incubation at 37 °C and 5% CO_2_, the samples were collected and fixed in 2.5% glutaraldehyde, followed by osmium tetroxide for 2 h at 4 °C. The fixed samples were subjected to gradient alcohol dehydration and critical point drying, and the surface was sprayed with gold. Scanning electron microscopy (SEM, Hitachi, Tokyo, Japan) was used to observe the cell morphology on the surface of each sample. All experiments were performed in triplicate.

### 4.6. Cytoskeletal Staining

Direct immunofluorescence staining was used to observe focal adhesion kinase (FAK). The BMMCs incubated on alloy samples for 3 days were fixed with 4% paraformaldehyde for 10 min, treated with 0.5% Triton-X for 20 min, and incubated with 5% BSA for 20 min to block nonspecific protein binding. The FITC-labeled FAK antibody was then incubated with the cells for 2 h. In addition, rhodamine-labeled phalloidin and 4′,6-diamidino-2-phenylindole (DAPI) were co-stained with F-actin and nuclei to localize the cell locations, and the FAK distribution was observed using fluorescence microscopy. All experiments were performed in triplicate.

### 4.7. ALP Secretion

The effects of the alloy samples on the osteogenic differentiation of the BMMCs were evaluated by detecting the alkaline phosphatase content in the cell culture medium. The BMMCs were seeded at a concentration of 1 × 10^6^/mL in 24-well plates containing XY-NiTi or XZ-NiTi. The cells cultured without alloys were used as a control group. After 1, 3, and 5 days of culture, 50 μL of the supernatant was collected. An ALP ELISA kit (Beyotime, Nantong, China) was used to detect the content of ALP in the supernatant. All experiments were performed six times.

### 4.8. RT-PCR of Osteogenic-Related Gene 

Bone marrow mesenchymal cells (BMMCs, passage 3) were seeded at 5 × 10^3^ cells/cm^2^ onto XY-NiTi, XZ-NiTi, or blank wells (control group) for 3 days. Total RNA was extracted using TRIzol reagent (1 mL/well; Invitrogen, Carlsbad, CA, USA), quantified via a NanoDrop 2000 (A260/A280 = 1.92 ± 0.15), and assessed for integrity by agarose gel electrophoresis (28S/18S rRNA ratio > 1.5). Reverse transcription was performed with 1 μg of RNA using the PrimeScript™ RT Reagent Kit (RR047A, Takara, San Jose, CA, USA) incorporating gDNA Eraser pretreatment (at 42 °C for 2 min) and random hexamer primers in a 20 μL reaction (at 42 °C for 15 min; at 85 °C for 5 s). qPCR amplification was conducted with a LightCycler 480 II (Roche, Basel, Switzerland) using SYBR Premix Ex Taq II (RR820A, Takara, Dalian, China) in 20 μL reactions containing 2 μL of cDNA, 0.2 μM of the primers, and the following cycling protocol: 95 °C for 10 min, and 40 cycles of 95 °C/15 s and 60 °C/1 min. Melt curve analysis confirmed the primer specificity (single peaks), and the amplification efficiencies (95–105%) were calculated from the standard curves. The controls included no-template (NTC) and no-reverse-transcription (NRC) reactions. The relative gene expression (*ALP*, *RUNX2*, *OPG*, and *OCN*) was normalized to GAPDH (geNorm M-value < 0.5) using the 2−ΔΔCt method, with data from five biological replicates (five technical replicates each) meeting an intra-assay CV of <5% and LinRegPCR efficiency correction. The primer design is listed in Table 2.

### 4.9. Statistical Analysis

The results are presented as the means ± standard deviations. The differences between the treatment groups were analyzed using one-way/two-way analysis of variance. Differences with *p* < 0.05 were considered statistically significant.

## 5. Conclusions

This study demonstrates that laser powder bed fusion (LPBF)-fabricated NiTi alloys exhibit anisotropic osteoinductive capabilities, with XY-NiTi outperforming XZ-NiTi in bone marrow mesenchymal cell (BMMC) adhesion and osteogenic differentiation (enhanced ALP activity, RUNX2, ALP, OCN, and OPG expression), likely attributed to its smaller grain structure. While limitations include the lack of direct comparison with other alloys and long-term in vivo validation, the findings advocate for prioritizing XY-oriented LPBF-NiTi in load-bearing prostheses to optimize osseointegration, offering critical guidance for improving orthopedic implant design and clinical outcomes.

## Figures and Tables

**Table 1:** Cell viability of the bone marrow mesenchymal cells cultured in leaching liquors of the XY-NiTi and XZ-NiTi alloys for two, four, or six days.

Time	Cell Viability	
	XY-NiTi	XZ-NiTi
Day 2	109.24%	93.42%
Day 4	105.05%	95.12%
Day 6	103.18%	103.52%

**Table 2:** Primer sequences of the genes.

Gene Subtype	Oligonucleotide Primers (5′–3′)
*ALP*	F: ATCGGACCCTGCCTTACC R: CTCTTGGGCTTGCTGTCG
*RUNX2*	F: ACTACCAGCCACCGAGACCA R: ACTGCTTGCAGCCTTAAATGACTCT
*OPG*	F: CAAATGTCCTCCTGGCACCT R: TTCACTGGTGTGCCAAGTGT
*OCN*	F: AGCCACCGAGACACCATGAGA R: AGCCACCGAGACACCATGAGA
*GAPDH*	F: CAATGACCCCTTCATTGACC R: TGGACTCCACGACGTACTCA

## Data Availability

All data generated or analyzed during this study are included in this published article.
